# CenoDerm vs. Fascia lata for the Prevention of Dorsal Nasal Irregularities in Rhinoplasty

**Published:** 2016-07

**Authors:** Alireza Mohebbi, Roghayeh Hamidian, Seyed-Behzad Poosti, Seyedeh-Simindokht Hosseini

**Affiliations:** 1 *Otorhinolaryngology and Head and Neck Research Center, Hazrat Rasoul Akram Hospital, Iran University of Medical Sciences, Tehran, Iran. *; 2 *Department of Otorhinolaryngology and Head and Neck, Hazrat Rasoul Akram Hospital, Iran University of Medical Sciences, Tehran, Iran. *; 3 *Eye Research Center, Farabi Eye Hospital, Tehran University of Medical Sciences, Tehran, Iran.*

**Keywords:** Acellular dermis, Fascia lata, Rhinoplasty

## Abstract

**Introduction:**

Dorsal nasal irregularity is a complication of rhinoplasty surgery, mostly seen in patients with thin skin. Acellular dermis (CenoDerm) and homologous fascia lata covering the nasal bone cartilage structure have been used to achieve a smooth surface. In this study, we aimed to investigate clinical outcomes using these two materials.

**Materials and Methods:**

After a standard rhinoplasty procedure, a layer of the acellular dermis or homologous fascia lata was placed in the pocket of the dorsum. Patients were evaluated for clinical outcomes at 3, 6, and 12 months after the procedure.

**Results:**

Forty-two of 68 patients completed the follow-up period. Patient satisfaction was higher in the homologous fascia lata group. Similarly, nasal dorsum inspection and palpation results were better in the homologous fascia lata group compared with the CenoDerm group but was significant in palpation (P=0.00). There was no complete absorption in the homologous fascia lata group 6 months after surgery (P= 0.04 vs. CenoDerm) but no significant difference was observed at 12 months.

**Conclusion:**

Homologous fascia lata is better than acellular dermis in preventing dorsal nasal irregularity after rhinoplasty in thin-skinned patients.

## Introduction

Irregularities and adhesions to the underlying structures in the nasal dorsum may be seen after rhinoplasty surgery or nasal trauma. Dorsal irregularity is reported to occur in 7–10% of all primary rhinoplasties, requiring revision surgery. Thin skin is the major risk factor (1). Grafts are available in the forms of autologous, homologous, and allogenic tissues that are used for both the prevention and correction of dorsal nasal irregularities, particularly in the thin-skinned patients. Increased risk of infection, extrusion, and late inflammation are all drawbacks of alloplastic materials (2). Autologous fascia is used for onlay grafts at the radix and dorsum; however harvesting autologous fascia requires an additional incision and procedure (3). Homologous fascia, such as Tutoplast-processed fascia lata (TPFL), is not associated with harvesting morbidity or additional operating time (3). Many clinical studies have shown the stability of fascia grafts and their good tolerability after transplantation. However, differing absorption rates have been reported. Based on previous studies, acellular dermis is easy to use and does not change over time. In addition, acellular dermis is soft, thin, pliable, and natural (4).

Few clinical studies have investigated the material absorption rate of fascia grafts, and those that are reported are insufficient because of limitations of objective assessment of the materials. To date, there have been no studies to compare the clinical outcome of a homologous fascia lata graft vs acellular dermis graft for the prevention of dorsal nasal irregularities. In this randomized clinical trial, acellular dermis and homologous fascia lata grafts are used as onlay grafts, and their effectiveness in the prevention of nasal dorsal irregularities after a rhinoplasty procedure is investigated. It was considered that if these products demonstrated good efficacy, this may provide a viable new option for the surgeon and patient as the graft is easy to use, does not require harvesting procedures, and is not subject to allogenic complications.

## Materials and Methods

*Subjects: *This study was conducted in a private clinic between 2013 and 2015. The trial was registered at the Iranian Registry of Clinical Trials with registration number IRCT 20140 41717312N1. 

Thin-skinned patients over 18 years of age, with a dorsal nasal hump without modification of the radix, were enrolled for rhinoplasty. The assessment of thin skin was confirmed by two expert surgeons on inspection and palpation. After a thorough explanation of the materials and methods, patients who agreed to become a study case in this randomized clinical trial were recruited, and all provided signed written informed consent. Patients with a previous history of rhinoplasty or other surgery on the nose, nasal trauma and fracture, diabetes mellitus, immunologic diseases, or vascular and hemorrhagic disease were excluded. Patients were divided into two groups of 34 using a permuted block randomization method, and remained blinded with respect to the technique performed on them. Surgeries were performed by the same surgeon in all cases (first author). Because of the nature of the intervention, the surgeon could not be blinded with respect to the procedure. To prevent possible bias, the surgeon was committed to perform the procedure in an exactly randomized manner, and he was not permitted to change the surgical plan for any individual in the operating room. 

*Graft Processing*

The following steps were followed for graft processing. 1) Case selection; 2) Tissue harvesting and processing in a sterile room; 3) Tissue assessment using several microbiologic and serologic tests; 4) Delipidization in both grafts and hair removal in the case of CenoDerm; 5) Rendering the graft acellular; 6) Freezing of the graft at-60-80°*C* and drying (<0.5 water activity); 6) Gamma irradiation.

*Surgical Procedures*

After elevating the skin flap and removal of the dorsal hump without modification of the radix, the requirement for correction in each patient was evaluated. Then, at the end of surgery, a layer of acellular dermis (Ceno Derm; Tissue Regeneration Corporation, Tehran, Iran) or homologous fascia lata (Fascia Lata;Tissue Regeneration Corporation, Tehran, Iran), with a thickness of 0.6–0.9 mm and measuring 20×40 mm, was placed in the pocket of the dorsum.

*Assessment of Surgical Outcome*

**Fig 1:**
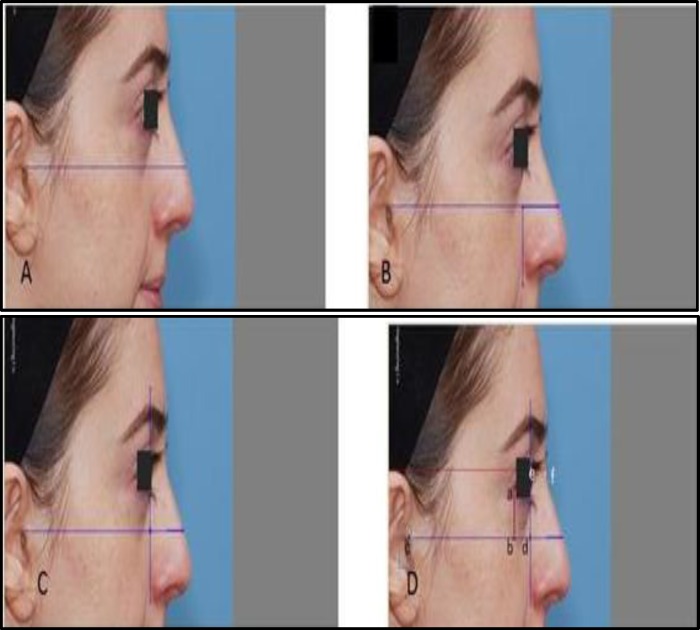
a) Standard Frankfort horizontal plane was delineated; B) Alar-facial line was delineated vertically; c,d) Dorsal radix height; (e-f) was measured by drawing a parallel line to Frankfort plane and a vertical line to alar – facial line, Then** a–b** and **c–d** distances were delineated and matched on the post-operation photographs, and distance of **e–f** on the post-operation photographs was measured

Standardized color photographs of each patient were taken before and after the rhinoplasty procedure and 3, 6, and 12 months after the procedure. Patients were evaluated for signs of infection, seroma formation, extrusion, and migration of materials by a physician who was not involved in the treatment. To assess absorption rate as the primary outcome of the study, the dorsal height was measured using Teb Negar Cosmetic Simusurge computer software,6 and 12 months after surgery. Measurements were performed on right lateral photographs (Fig.1). 

 Next, the preoperative dorsal height was subtracted from the values measured at 6 and 12 months. Six groups were defined based on graft thickness: No absorption (thickness ≥ 0.6 mm); Low absorption (0.45 mm ≤ thickness<0.6 mm); Moderate absorption (0.3 mm ≤ thickness < 0.45 mm); High absorption (0.15 mm ≤ thickness <0.3mm); Very high absorption (0< thickness< 0.15 mm); Complete absorption (≤ 0 mm).

Twelve months after surgery, inspection and palpation of the nasal dorsum to identify irregularities was performed by a physician blinded with respect to the technique used in each patient. Based on this assessment, cases were classified into the following groups: very high irregularity, high, medium, low, very low, and no irregularity.

At this 12-month visit, patient satisfaction with respect to irregularities of the nasal dorsum was also addressed using previously modified written forms based on five-point Likert scales (scores from 1 to 5). Patients were asked to rate satisfaction as very low, low, medium, high, or very high according to above scores by the same physician, and the responses were recorded.

*Statistical Analysis*

The data collected were analyzed by SPSS software 20, for quantitative variables between the two groups. Mean values and frequencies were estimated in each group. A t-test or Mann-Whitney test was used. The chi-square test or Fisher's exact test were used to analyze qualitative data. A P<0.05 was considered statistically significant.

## Results

**Fig 2:**
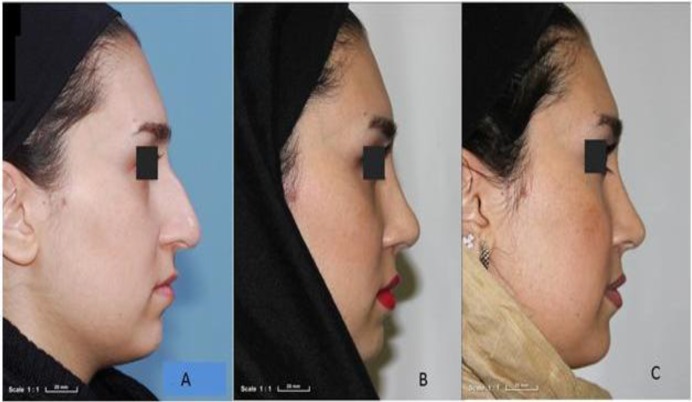
Patient from homologous fascia lata group; no irregularity (6 scores) on inspection and palpation; A) pre-OP; B) 6 months; C) 12 months

Of 68 patients who underwent rhinoplasty surgery from March 2013 to 2015, 42 completed the follow-up period. There were 18 cases in the fascia lata group, of whom three (16.7%) were male; the acellular dermis group included 24 patients, of whom four (16.7%) were male. The age range was 25–55 years (mean, 34.94±9.04 years) in the homologous fascia lata group and 22–48 years (mean, 31.25±6.93 years) in the acellular dermis group. No significant difference was found between the mean ages in the two groups (P=0.142). At Month 12, the results of inspection and palpation were better in the homologous fascia lata group compared with the acellular dermis group. On inspection in the homologous fascia lata group, 12 cases had no irregularity and six had a very low irregularity. Findings on palpation in the homologous fascia lata group showed nine patients had no irregularity and nine had very low irregularity (Fig.2). 

**Fig 3:**
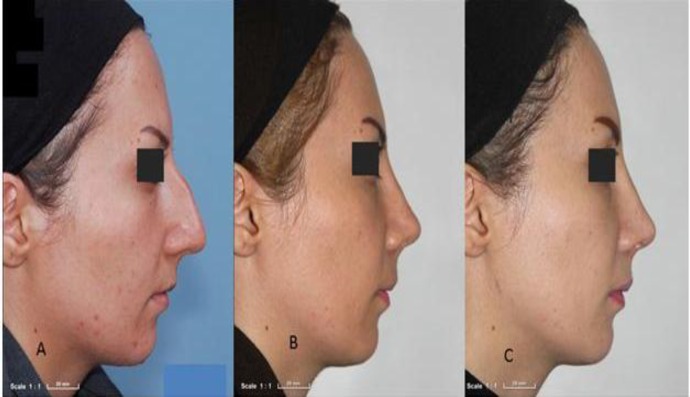
Patient from acellular dermis graft group; low (4 scores) dorsal nasal irregularity on inspection and moderate (3 scores) on palpation. A) pre-OP; B) 6 months; C) 12 months

In contrast, on inspection in the acellular dermis group, the numbers of patients with no, very low, low, moderate irregularity were 12, two, six, and four, respectively. On palpation in this group, two, eight, six, four, and two patients, respectively, had no, very low, low, moderate, and high degrees of irregularity (Fig.3).

**Fig 4:**
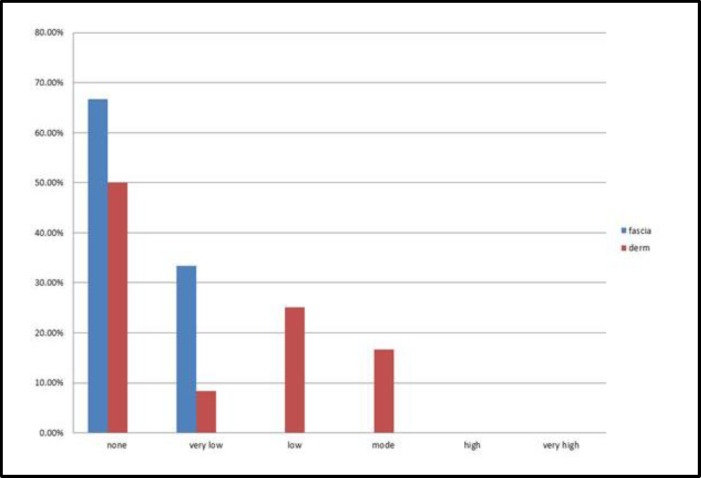
Inspection findings (dorsal irregularity) after 12 months of surgery; no significant difference between the two groups (P=0.06

**Fig 5:**
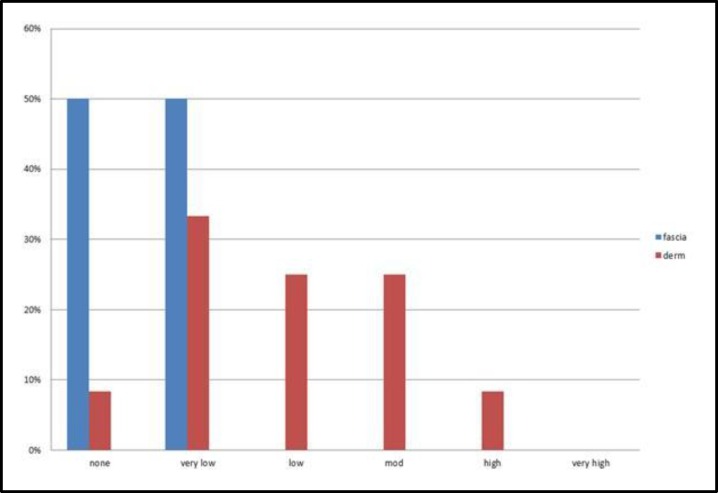
Palpation findings (dorsal irregularity) after 12 months of surgery; significant difference between the two groups (P = 0.00

These differences were statistically significant with respect to palpation, but not inspection (Figs 4,5).

Patient satisfaction was evaluated at Month 12. In the homologous fascia lata group, of 18 patients, six had a score of 5 and 12 had a score of 4. Among the 24 patients of the acellular dermis group, 11 declared a score of 4, 12 had a score of 3, and one had a score of 2. No scores of 5 were reported in the acellular dermis group. This improvement in the homologous fascia lata group was statistically significant according to the non-parametric test analysis (P=0.00). The correlation coefficient test showed that there was a statistically significant correlation between the results of dorsum palpation and inspection in both group (P=0.001). Patient satisfaction and physician palpation showed a statistically significant correlation in the acellular dermis group (P=0.009), but no correlation was found in the homologous fascia lata group (P=1.000). In comparison, the correlation between patient satisfaction and physician inspection was statistically significant in the homologous fascia lata group (P=0.024), while no such correlation was found in the acellular dermis group (P = 0.317). The results of absorption of the materials are shown in Table 1.

**Table 1:** Comparison of absorption rate in the acellular dermis and fascia lata graft, 6 and 12 months after surgery

		**None**	**Low**	**Moderate**	**High**	**Very high**	**Complete**
Fascia Lata	6m	6 (33.3%)	3 (16.7%)	3 (16.7%)	3 (16.7%)	3 (16.7%)	-
	12m	-	5 (27.8%)	-	3(16.7%)	6 (33.3 %)	4(22.4%)
Ceno- Derm	6m	3 (12.5%)	2 (8.3 %)	6 (25 %)	4(16.7%)	5 (20.8 %)	4(16.7%
	12m	3 (12.5%)	-	-	6 (25 %)	6 (25 %)	9 (37.5 %)

**Fig 6:**
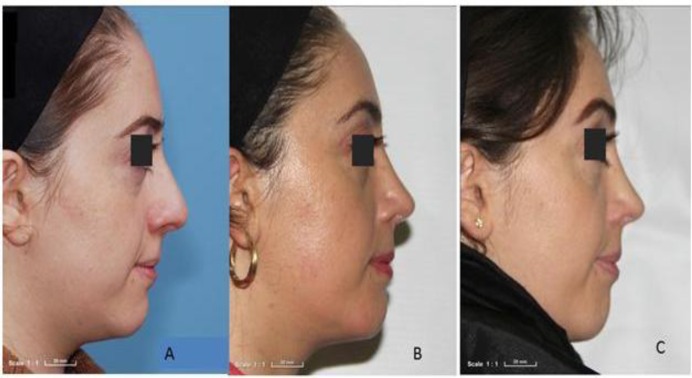
Patient from fascia lata graft group without absorption in 6 months and with low absorption at 12 months; A) pre-OP; B) 6 months; C) 12 months

**Fig 7:**
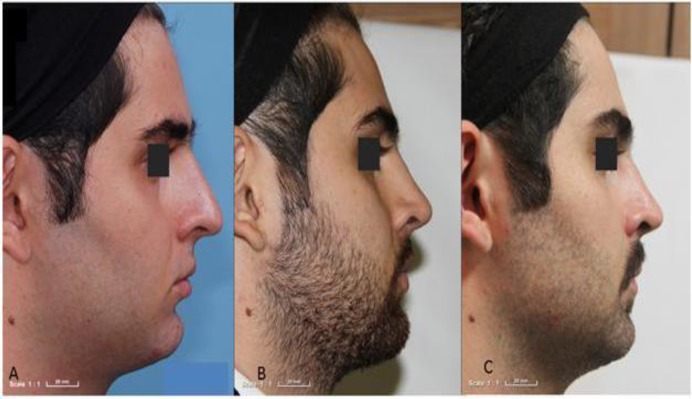
Patient from acellular dermis graft group; very high absorption at 6 months and complete. absorption at 12 months; A) pre-OP; B) 6 months; C) 12 months

There was no complete absorption in the fascia lata group at 6 months after surgery, and a statistically significant difference was found between the two groups at this time(P= 0.04). However, at the end of the Month 12, complete absorption occurred in four cases (22.2%) in the fascia lata group, with no significant difference between the two groups (P= 0.386) (Figs 6,7).

## Discussion

Rhinoplasty operations are among the most common aesthetic procedures. Palpable dorsal irregularities are among the complications of rhinoplasty, and are more common in patients with thin skin (1). This problem can be a consequence of implant materials such as autografts, homografts, and alloplasts (2). In patients with thin skin, graft materials are occasionally used in primary rhinoplasty procedures for the prevention of visible dorsal nasal irregularities. Cartilage, dermis, temporal fascia, superficial musculoapo- neurotic system (SMAS), Vicryl mesh, gelatin film, polytetrafluoroethylene, Surgicel-wrapped diced and crushed cartilage, tensor fascia lata, and acellular dermis are among the most commonly used materials to correct contour deformities and provide a subcutaneous pad (1). Acellular dermis has been used in rhinoplasty for dorsal nasal irregularity, dorsal augmentation, and septal perforation. It has also been used for abdominal wall reconstruction in hernias, and in gum and lip augmentation. The complication rate associated with acellular dermis graft has been reported as more than 20 percent in hernia repair surgery; similarly, complete absorption in lip augmentation has been reported (5-10). Similarly, homologous fascia lata has been used in orthopedic surgery as well as abdominal and plastic surgery. Ophthalmic, gynecologic, and urologic surgeries are other uses, as well as use in stress incontinence, correction of congenital ptosis, and correction of facial nerve paralysis (11,12). The current study is the first designed to compare the outcomes of these two materials for the correction of dorsal nasal irregularities in rhinoplasty surgery.

In other studies, the brand of acellular dermis used has tended to be AlloDerm. However, Iran has only recently launched production of this material, and the experience and research in this field is still very scarce. In our study the brand of acellular dermis used was CenoDerm. 

In this study, patients in neither group had complications such as infection, migration of material, extrusion, or seroma formation in the follow-up period. This is consistent with previous data (1). Only in one patient in the Gryskiewicz study was partial extrusion of AlloDerm reported. In this patient, the AlloDerm graft was rolled to form six layers and placed using an endonasal approach. More recently it is understood that stacking the grafts in layers is preferable to rolling the grafts to prevent postoperative migration (13).

At the Month 6 postoperatively, there was no case of graft absorption in fascia group, but in at Month 12, it occurred completely in 16.7 percent of the patients. 

In the 2007 study by Ju Jang Y et al. designed to investigate Tutoplast-processed fascia lata for nasal dorsal augmentation in rhinoplasty, 69 patients were followed by photographs for 12 months after surgery. In 41 patients, fascia lata without other material combination was used, and complete absorption occurred in 4.3% percent of this group (14). In our acellular dermis group, complete absorption was detected at both 6 and 12 months; whereas after 12 months, it occurred in the fascia group only. A number of studies have been performed on the absorption rate of acellular dermis used for nasal dorsal irregularities. In the current study, quantitative parameters were used to describe the occurrence of absorption. However, in other studies, biopsy, pathology, and counter deformity assessment based on photographs have been used. In an animal study, pathology was used to assess graft absorption. This method is invasive and not practicable for our patients (14). In another study, contour deformity on photography was used without explanation of the assessment and measurement used. In this study, graft absorption was shown using quantitative parameters (13). The method used in one study concerning lip augmentation involved use of alar-base to lip point distances (9). The methodology used in our study was thus based on these studies, and in particular on the last one mentioned. 

In a prospective clinical animal study in rabbits, the absorption rate of acellular dermis for nasal dorsal irregularities was noted in 29.75% of cases. This result was achieved by biopsy 4 months after surgery (1). In another animal study investigating nasal dorsal irregularities, complete absorption of the acellular dermis 4 months after the graft was reported based on biopsy evaluation (15).

In a study in 2005, 25 secondary rhinoplasty patients who underwent acellular dermis graft were followed for 2 years or longer. Long-term results showed minimal absorption. Absorption was most common over the bony dorsum, and was overall estimated at 20–30% of cases; approximately absorption occurred in 10–15% of the patients over the tip. An evaluation was performed by means of photographic analysis (13).

These studies demonstrate the different absorption rates via various estimation methods; although our results are not consistent with them. Of course, biopsy is a more precise, reliable method of evaluation, but invasiveness is a highly important issue and is not acceptable in a live patient after cosmetic plastic surgery. As understood now, the efficacy of these materials is based on prevention of adhesion of the skin to the bone underneath (16). 

Better outcomes in the homologous fascia lata graft group can be associated with its non-absorption after 6 months and a slower absorption rate than the acellular dermis graft group between 6 and 12 months after surgery. This slower rate provides a sufficient time period for wound repair without any adhesions established between bone and the skin covering it, that are supposed to be the causes of dorsal nasal irregularity. Presence of skin edema might after 4 months potentially causes difficulty in assessment, and evaluation was therefore performed at Month 6 in our study. Based on previous studies, there was no relationship between absorption and the number of layers of the graft used; however, we used monolayer material in both groups (4). 

In a 2014 study by Dong Won Lee et al, two and three layers of acellular dermis were utilized for dorsal nasal augmentation in 20 patients in two groups. The degree of angiogenesis and the synthesis of collagen and elastin fibers were evaluated over 40 days. Thickness decreased in both groups, but there was no statistically significant difference in volume (17).

Poor patient cooperation for fulfillment of the follow-up sessions is an important issue in a group of cosmetic surgery patients that are highly satisfied with their outcomes. One patient from fascia group was excluded due to diagnosis of Wegner disease during her follow-up. Some patients were excluded because of no regular follow-up, with incomplete information and photography. A phone call was made to evaluate many of the patients that did not arrive at the Month 12 assessment, and reasons provided included being far from the clinic because they live in another city of Iran, or traveling abroad. A busy work schedule was the reason among other patients. No response to our call phone led to incomplete follow-up and a lack of information in other patients, perhaps because the individual was satisfied with the shape of his or her nose. Despite providing consent for a 12-month follow-up, these patients did not return for objective evaluation and we were not able to analyze their data. Patient follow-up was worse in the fascia lata group, possibly due to better outcomes in this group. 

Longer-term follow-up evaluations of more than 12 months in a larger study population which included a control group who did not undergo any graft procedure would lead to more reliable results.

## Conclusion

Despite the limitations of this study, use of homologous fascia lata appears preferable to acellular dermis graft in order to achieve lower rates of dorsal nasal irregularity in standard rhinoplasty surgery. 
